# Perceptions on the Use of Wearable Sensors and Continuous Monitoring in Surgical Patients: Interview Study Among Surgical Staff

**DOI:** 10.2196/27866

**Published:** 2022-02-11

**Authors:** Meera Joshi, Stephanie Archer, Abigail Morbi, Hutan Ashrafian, Sonal Arora, Sadia Khan, Graham Cooke, Ara Darzi

**Affiliations:** 1 Department of Surgery & Cancer Imperial College London London United Kingdom; 2 Chelsea and Westminster Hospital National Health Service Foundation Trust London United Kingdom; 3 Department of Infectious Diseases Imperial College London London United Kingdom

**Keywords:** staff feedback, interview, sensors, continuous monitoring, mobile phone

## Abstract

**Background:**

Continuous vital sign monitoring by using wearable sensors may result in the earlier detection of patient deterioration and sepsis. Few studies have explored the perspectives of surgical team members on the use of such sensors in surgical patients.

**Objective:**

This study aims to understand the views of surgical team members regarding novel wearable sensors for surgical patients.

**Methods:**

Wearable sensors that monitor vital signs (heart rate, respiratory rate, and temperature) continuously were used by acute surgical patients. The opinions of surgical staff who were treating patients with these sensors were collated through in-depth semistructured interviews to thematic saturation. Interviews were audio recorded, transcribed, and analyzed via thematic analysis.

**Results:**

A total of 48 interviews were performed with senior and junior surgeons and senior and junior nurses. The main themes of interest that emerged from the interviews were (1) problems with current monitoring, (2) the anticipated impact of wearables on patient safety, (3) the impact on staff, (4) the impact on patients overall, (5) potential new changes, and (6) the future and views on technology.

**Conclusions:**

Overall, the feedback from staff who were continuously monitoring surgical patients via wearable sensors was positive, and relatively few concerns were raised. Surgical staff members identify problems with current monitoring and anticipate that sensors will both improve patient safety and be the future of monitoring.

## Introduction

The failure to recognize and respond to patient deterioration is a major cause of morbidity and mortality and is predominantly caused by human monitoring factors [1-3]. Patients undergoing major surgery are at risk of life-threatening complications. The earlier recognition of deterioration can improve survival and may also reduce patients’ length of hospital stay and the need for higher acuity care [4]. The detection of deterioration occurs by measuring vital signs routinely every 4 to 6 hours or more frequently in patients who are identified as unwell. Until now, continuous monitoring has only been feasible in higher dependency settings via invasive methods. However, the latest lightweight sensors have the potential to be used for the continuous monitoring of all hospitalized patients.

Previous studies have reviewed the reliability of wearable devices [5,6], but further research is needed to understand the performance of these devices [7] and staff perspectives. There are very few studies that have reviewed staff experiences [8]; a systematic review of patients’ and staff’s experiences with wearable sensors found a lack of high-quality studies in this subject area [7]. The studies reviewed had a small sample size (most had less than 20 participants), and their reporting of the research process was limited [7]. Further, the end user outcomes were often a secondary aim rather than the primary focus.

In order to assess the acceptability of wearable sensors, the opinions of all who use technology must be reviewed. We anticipated that feedback may be dependent on role and that wearable sensors would have the greatest impact on nurses, as they are the ones who place sensors on patients and are the first to be alerted about deterioration. This study aims to conduct a comprehensive exploration of interdisciplinary staff views on wearable sensor technology for surgical patients.

## Methods

### Study Design

Semistructured interviews were held for a subgroup of surgical health care staff to explore their experiences with and perceptions of wearable sensors in detail.

### Ethical Approval

Ethical approval was granted by Yorkshire & The Humber - Leeds East Research Ethics Committee (reference number: 17/YH/0296).

### Participants and Setting

All members of staff had been treating patients wearing a wearable sensor in the wearable patch at West Middlesex University Hospital—a busy hospital located in northwest London that serves an ethnically diverse population. Interviews were conducted starting in March 2018 and were discontinued once preliminary thematic saturation had been attained [9,10].

### Sensium Sensor

Acutely unwell patients admitted to surgical wards were offered the Sensium Vitals (The Surgical Company) wearable sensor in addition to standard ward vital sign monitoring by nurses. The sensor is lightweight; measures heart rate, respiratory rate, and temperature every 2 minutes; and has a battery life of 5 days. For longer hospitalization periods, an additional sensor is required. Sensor data flow from the sensor to a web-based server via a bridge before they are sent via Wi-Fi to mobile apps and smartphone devices.

**Figure 1:**
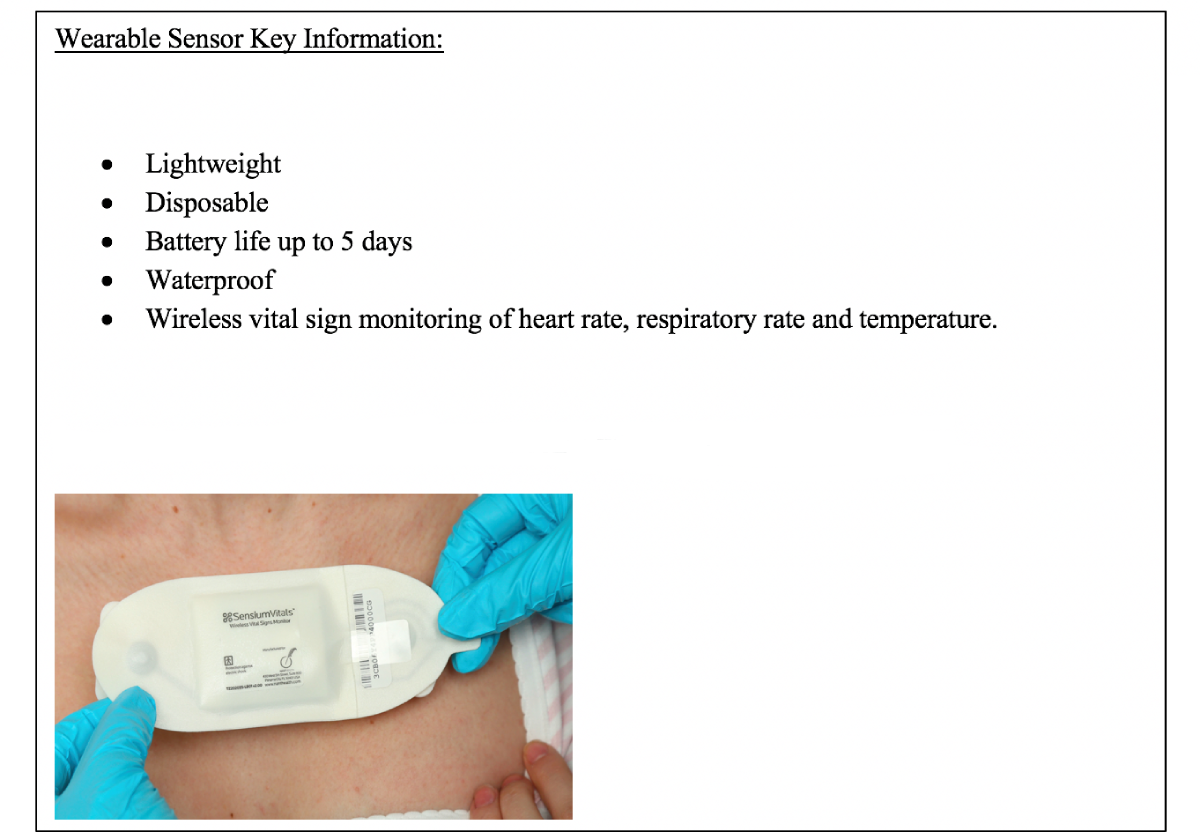
Properties of the wearable sensor. The picture shows one of the sensors being worn on a patient's chest. The image was reproduced with permission from Sensium (Abingdon, United Kingdom).

In Figure 1, the sensor placement on a patient’s chest can be seen. The sensor was placed by either the trained health care professionals who were looking after the patients or the research team. The patch was attached to the anterior chest wall by using 2 standard disposable electrocardiogram electrodes (Red-Dot2560; 3M Company). Medical tape was used to ensure that the temperature probe was secured in the axilla.

A plastic strip was pulled to activate the sensor. The sensor recorded in a sequential, cyclical, 2-minute fashion. A predictive strategy was used to calculate heart rate based on the RR interval [11]. The RR interval is the time that elapses between 2 successive R waves in a QRS signal on an electrocardiogram. This approach has been described previously [12, 13]. The individual RR intervals from the electrocardiogram strip were rank ordered, and the median value was taken as the average heart rate. Impedance pneumography was the technique used by the Sensium sensor to measure respiratory rate. This is a common technique that is used to measure a person’s breathing rate [14]. Impedance was measured through superficial electrodes. The impedance measures both the respiratory volume and the respiratory rate via the relationship between depth and thoracic impedance change [15]. The respiratory rates were derived from changes in the impedance of the thorax due to inhalation and exhalation. A very small current (iK) was injected through the electrocardiogram electrodes. The thoracic impedance changes were detected as variations in the voltage (V) measured at the electrocardiogram electrodes. Inhalation (peak resistance) and exhalation (trough resistance) were detected from a 60-second segment of the impedance pneumography waveform to calculate a median respiratory rate. Temperature was measured by using a calibrated thermistor (ie, a temperature-sensitive resistor; Braun ThermoScan PRO 6000; Welch Allyn Braun), which was placed in patients’ axillae. Individual vital sign readings were measured and processed in time order.

**Figure 2:**
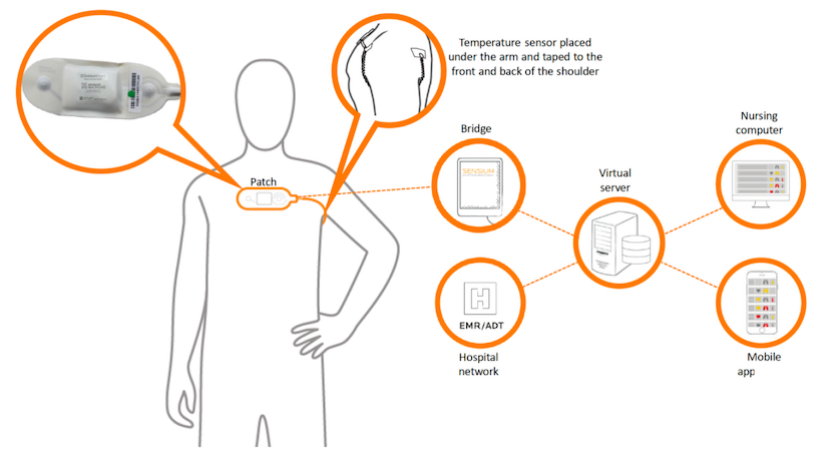
Sensium wearable sensor data transmission to the server and then to mobile apps or computers. The image was reproduced with permission from Sensium (Abingdon, United Kingdom).

Once the sensor captured physiological data, it used an algorithm, which was stored in a microchip, to process the data. This microchip had a built-in processing unit that transmitted the average values for heart rate as beats per minute and those for respiratory rate as breaths per minute to the nearest bridge. These data were then transmitted to the central server [11], thus allowing digital alerts to be sent to health care staff through smartphones or electronic health records (Figure 2).

Data security is of paramount importance when using wearable technology. The Sensium system is International Organization for Standardization 27001 compliant (information security management), safe, and secure. The Sensium patches are uniquely identified by means of a machine-readable serial number, and these numbers can be matched to a patient ID band on the Sensium server via a bar code scanner. No patient identifiable information was being communicated from the Sensium patch to the Sensium bridge; just the serial numbers of the devices and values for heart rate, respiratory rate, and temperature were transmitted. Once the information was transferred from the Sensium bridge to the secure Sensium server, only then were the values from the patch put into context with patient identifiable demographic information, usually with the help of a patient administration system. The Sensium patch transmits data to the Sensium bridge every 2 minutes and receives a positive acknowledgement back from the Sensium server when the data have been received. If the patch is out of range of a Sensium bridge or if no acknowledgement is received from the server, the patch continues to attempt communication until it is successful. The Sensium patch stores up to 3 hours’ worth of data locally and passes this information to a Sensium bridge once it is back in range.

Once the wearable sensor had been used in the surgical wards for 3 months, semistructured interviews with staff members were conducted. At this stage, the sensor did not alert staff members in real time, and sensor data were only available to the research team.

A wide range of staff participants were sampled, including junior and senior nurses as well as interns/attendings (senior surgeons), to ensure that an interdisciplinary assessment was conducted [16]. All staff members interviewed had applied the wearable sensor, undergone training, or managed a patient that had worn the sensor. The sensor did not alert staff in real time and was not part of day-to-day care; it was only used for research purposes.

### Qualitative Data Analyses

Semistructured interviews were conducted to allow for the in-depth exploration of staff perceptions. The interview guide was developed through an extensive literature search and previous piloting. Face-to-face interviews were conducted by the lead researcher (MJ)—a surgical resident with no personal or professional ties to the interviewees.

Interviews were audio recorded and transcribed verbatim. Interview data were analyzed by using thematic analysis [17]. All transcripts were reviewed multiple times and coded by a second independent researcher (AM); any discrepancies in thematic codes were discussed until agreement was reached.

## Results

### Staff Characteristics

A total of 48 interviews were performed with 12 senior surgeons/attendings (experience: mean 19 years; range 12-44 years), 12 junior physicians (experience: mean 2 years; range 1-5 years), 12 senior nurses (experience: mean 14 years; range 5-20 years), and 12 junior nurses (experience: mean 9 years; range 1-20 years). The staff demographics can be found in Multimedia Appendix 1.

**Figure 3:**
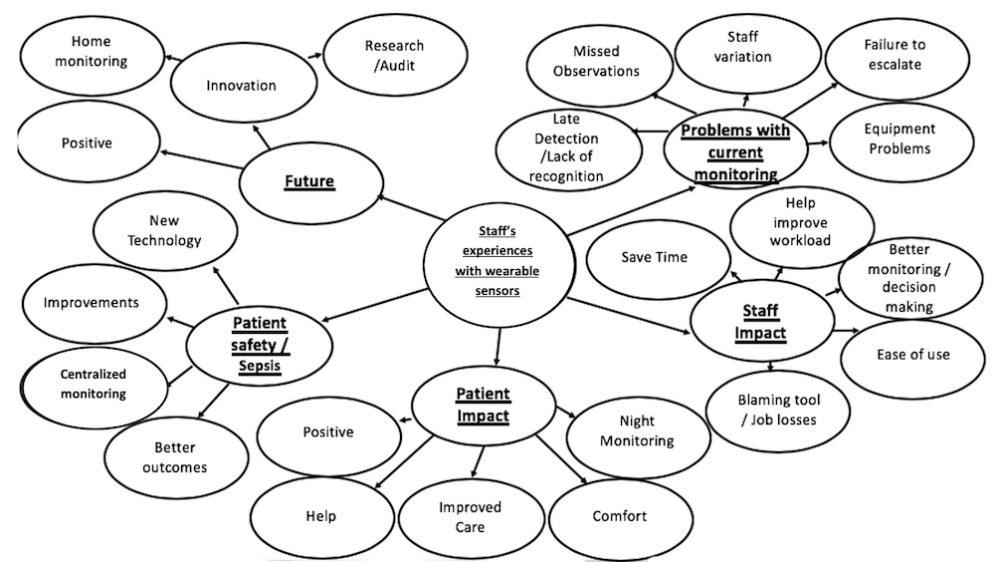
Themes and subthemes from the thematic analysis.

Figure 3 details the themes and subthemes resulting from the thematic analysis; these are expounded in the following sections and illustrated with verbatim quotations.

### Problems With Current Monitoring

Staff identified problems with current monitoring; patient deterioration was often identified late. Investigations into adverse outcomes or significant harm in patients found that patient deterioration signs were present several hours before they were detected clinically:

Deteriorating patients are often picked late, despite all of the scoring systems that we have. Actually, when you look back at cause analysis and a lot of deterioration, there's always a sign there that was present hours before they were picked up.

Senior surgeon #11

Staff suggested that patient deterioration was sometimes overlooked between observations and compounded by missed observations. It was noted that variations in the training and education of junior staff might result in delayed or missed observations. Agency staff were also associated with missed observations:

We had a healthcare assistant who was agency, doesn’t really work here much, where we thought things were up to date and when we went back to check, an observation hadn’t been done, that patient had spiked [temperature], so obviously there’s that time period in between we could have been acting sooner.

Senior nurse #11

Another concern was a lack of the recognition of an unwell patient. Senior surgeons identified this as being a common problem with inaccurate or incomplete patient assessments and a failure to escalate, even in patients with sepsis:

The lack of recognising the development of sepsis and the lack of recognising deteriorating NEWS [national early warning] scores is a relatively common theme amongst serious incident patients.

Senior surgeon #2

Staff thought that a lack of confidence might result in a failure to escalate; the greater and sustained education of staff members may combat this issue:

Part of the whole sepsis story, it’s all about education of staff, being able to have the confidence to institute treatment, I see it so often where the patient’s deterioration is accurately monitored but then nothing’s done about it.

Senior surgeon #8

Practical reasons for failures in monitoring and patient deterioration detection included staff shortages and the resulting additional demands placed on existing staff members. Staff stated that during night shifts, the duties of both nurses and physicians were often stretched. This was particularly true for staff who covered other people’s allocated breaks:

If on my break or during the night-time it’s only one HCA for two bays. It’s meaning if I’m on my break definitely nobody is doing [observations] instead of me.

Junior nurse #12

Nursing staff also cited faulty equipment and the lack of enough observation machines as compounding problems.

### Patient Safety

All staff believed that using wearable sensors and conducting continuous monitoring would improve patient safety. They anticipated that wearable sensors would identify unwell patients earlier and result in a reduction in error:

I think it’s really good that it’s happening a lot quicker and obviously using technology so that the nurses can be alerted if something sort of is going wrong so that even the more junior nurses have that sort of prompt.

Junior physician #5

Staff welcomed the use of digital alerts and felt that they would provide encouragement for raising an alarm should a problem arise:

It’s an element of probably giving people I suppose empowerment is quite a good word, but it gives them more confidence to make decisions.

Senior surgeon #8

Staff stated that although observation charts in most hospitals are still kept at the patients’ bedsides, centralized monitoring allowed all staff, regardless of their location, to identify unwell patients:

Like, if you are not physically there, present, and you can see anywhere in the screen if the patient is unwell or is deterioration in the patient observation.

Senior nurse #3

Staff also believed that the abundance of extra data captured allowed for further interpretation through trend analysis rather than just a single set of observations taken at 1 time point:

Would be quite interested to see trends in things like their heart rate for example, when they are recovering from an operation, it might give them peace of mind.

Junior physician #12

Staff felt that the new technology would be more accurate than their current monitoring technology and could be used to reduce variability when taking observations. In particular, the staff highlighted the sensor’s ability to detect respiratory rate as a great strength:

Well, we were discussing about the respiratory rate, it would be great to have a way of actually accurately measuring respiratory rate.

Junior physician #7

Faster detection, escalation, and treatment were also expected to result in better care and overall reductions in morbidity and mortality. Many staff highlighted the importance of the sensor in the timely identification of sepsis:

Now we have a patient with sepsis, and we didn’t find out at the beginning. With this machine maybe we should get the result before that, so that’s why it’s much better than to find that later in every four hours.

Junior nurse #12

In addition to the sensor’s potential benefits in surgery, staff acknowledged that other high-risk patient groups (eg, older patients) might benefit from wearable sensors and that such sensors improve the overall profile of patient safety, as they can help with making suggestions for a culture change. For instance, senior surgeon #11 said, “It’s also a cultural change of actually acting on the abnormalities earlier rather than later.”

### Impact on Staff

Overall, staff thought that the new technology would positively benefit those working in a surgical setting and expressed their optimism about the sensor’s potential to save time, particularly in settings where the demands are great. Junior physician #1 said, “It also saves doctors time as well in the sense that they know what’s appropriate and what they actually need to go.” Nursing staff also talked about how the sensors helped them to prioritize patients and allocate care more effectively:

It gives us an idea of which patients we need to be looking at, more promptly, who we need to be directing the nurses to, who we need to be sort of escalating more quickly.

Senior nurse #8

Another crucial strength of wearable sensors that was identified was the ease of use and interpretation. For example, senior surgeon #8 said, “It’s not difficult. Stick it on, plug it in.” However, although the impact of wearable sensors on staff workload was generally very positive, some junior physicians raised concerns that the workload of nursing staff may increase if too many alerts are generated:

When the nursing staff are busy, and they are having to deal with more alerts and things like that. If it is going off quite a bit, it is going to be difficult to triage, it might end up having to have a protocol for it I think.

Junior physician #11

Other junior physicians voiced concern about the use of the new technology in incident investigation and its potential use in litigation:

It may become a blaming tool because it sort of you know, registered it as a problem, but then if it’s not been acted on, then it becomes somewhere where it’s more like a legal sort of thing and people could use it towards that.

Junior physician #3

The final concern among junior physicians was that if the technology is too effective, staff who conduct observations regularly may lose their job:

There’s always a new problem it might actually put some people’s job in danger for example Healthcare Assistants, there’s no need to do observations on a regular basis because this thing does it for you.

Junior physician #3

### Impact on Patients

Staff generally believed that wearable sensors would be more comfortable for patients than their current monitoring technology:

I never heard any patient complain, if they had any allergy or they found it uncomfortable, I never heard any patient complain about it.

Junior nurse #3

Staff also observed that patients could carry out their normal day-to-day tasks, like bathing. This complemented the discreet nature of the patch:

It doesn’t disturb patients much. Most of the ones that I've seen haven’t found that – they’ve even forgotten that it’s on them.

Senior surgeon #12

As well as being discrete, the sensor was described as being noninvasive. For example, staff reported that the sensors were particularly beneficial for monitoring patients at night, as they caused less sleep disruption than that caused by their current methods of monitoring.

### Changes

A total of 54% (26/48) of participants thought that no changes were needed. Although the size of the patch was not identified as being a problem and was far smaller than the size of the staff’s current monitoring technology, all participant groups predicted future miniaturization. Staff participants felt that more parameters should be added in the future to allow for more complete monitoring. Alternative sensors that could be placed on different parts of the body were suggested, such as wrist or necklace sensors. A change to the temperature wire was advised by some of the junior nurses.

### The Future

All staff believed that wearable technology was the future and would replace the current monitoring systems. Junior nurse #9 said, “I think the way the future is, everybody will be wearing them.” Staff generally thought that wearable sensors were a positive concept and were innovative tools. They welcomed wearable sensors in health care and felt a need for the technology. For instance, junior physician #10 stated, “100%. I think the concept is needed.”

Suggestions were made by physicians on the use of wearable sensors in monitoring patients at home following discharge:

Also if these could be rolled out to patients who actually go home, so they could monitor themselves at home, rolling it out into maybe a community setting might be something to consider in the future.

Junior physician #12

Many participants advocated for the use of monitoring in all patients and felt that this would improve the data quality in clinical and research settings. Further, staff acknowledged that trends in the data, when combined with clinical data, could be used to identify and monitor treatment strategies:

So, for instance, if we ever wanted to have a look and see how our treatments are working and what the actual timeline or timeframe is for vital signs to actually normalise, grab all that data and use it for audits as well?

Junior physician #10

## Discussion

### Principal Findings

To our knowledge, this is the first study that provides an interdisciplinary view of wearable sensors for surgical ward patients. All staff groups welcomed the use of wearable sensors and acknowledged deficiencies in current monitoring techniques. Several key themes were identified, including the need for new technology, patient safety, the impact on staff and patients, changes, and future use.

The need for changes to current monitoring methods was clearly identified by delays in identifying unwell patients and a failure to escalate, as reported previously in the literature [2,18-21]. Wider literature shows that clinical deterioration may present several hours prior to an adverse event, and this was identified by staff members [16]. The nighttime use of wearable sensors in particular was found to be a key strength.

Staffing concerns were raised, including the use of agency staff; variations in junior staff quality; and staff shortages, especially those that occurred overnight. Low numbers of registered nurses and high numbers of admissions per registered nurse have been associated with increased mortality during a hospital admission [22,23]. Problems with staff’s current observation technology were also identified; not enough observation machines were available. Staff members highlighted the idea that wearable sensor monitoring may bridge these gaps.

Continuous monitoring was thought to result in the earlier recognition of unwell patients, faster escalation, and faster treatment. Centralized monitoring platforms for the remote monitoring of patients’ vital sign information offered staff reassurance. The poor documentation of vital signs has been consistently found for current monitoring techniques, with respiratory rate being the vital sign that is often missed [24]. With current surgical wards being overstretched, any potential assistance from new technologies was embraced and was thought to improve prioritization, multitasking, patient safety culture, and the awareness of unwell patients. The practical applications of small sensors were identified. This was combined with the use of the new technology to allow for easier interpretation, improve documentation, and help with decision-making.

Junior physicians highlighted concerns of increased workload for the nursing staff. Alarm fatigue is a problem in hospitals where many digital alerts are being generated not only from electronic health records but also from other devices [25]. With frequent alarms, there is the chance that staff may become desensitized to them. Sensitive and specific alarms that correctly identify unwell patients must be generated [25]. Novel ways of interpreting such large quantities of data generated from wearable sensors may be required, and one suggestion is to use trend analysis platforms.

All staff felt that wearable technology positively benefits patients and improves care. The sensors were comfortable overall and were well tolerated by patients. In contrast to a previous study [19], staff thought that many patients had forgotten that they were wearing the sensor. However, some improvements were suggested, such as future miniaturization and the addition of other vital sign parameters. These results are similar to those of a previous study on a wrist-worn sensor [26].

### Strengths

This is the first study to provide an interdisciplinary view of new continuous remote monitoring technology. Staff participants were recruited from several surgical areas. A clear need for the technology was identified by all staff groups. Staff engagement was high, and feedback has been overwhelmingly positive. Staff also highlighted that patients found that the monitoring technology was comfortable, with many forgetting that they had been wearing the sensor. From our results, we can see that the device was easy to use, and for a continuous monitoring device to be widely adopted and be useful, it must be easy to use [27]. In addition, the modeling of clinical and biological data (partly through the use of wearable sensors) is advancing rapidly and is likely to be a useful adjunct in identifying and predicting physiological changes in critically ill surgical patients [28].

### Limitations

This study collected data from a single hospital, and thus the findings may not be generalizable to other hospital settings. Although the Sensium wearable sensor was used in this study, our findings on patient comfort and ease of use for staff may not be transferrable to other sensor technologies. In addition, the wearable patches in this study were being used for research purposes, and real-time alerting was not yet established. It may be appropriate to repeat the interview with staff members in the future after digital alerting is established to determine if their opinions change.

### Conclusion

Through our interdisciplinary approach, we found wearable sensors to be a useful tool for identifying unwell patients and improving patient safety largely through the earlier escalation of care and treatment. We hope that this technology will benefit both staff and patients. Although several limitations were identified, wearable sensors have been embraced by staff with the belief that the use of such sensors will be the future of health care.

## Appendix

Multimedia Appendix 1 The demographics of staff interviewed and years of experience.

## Abbreviations

NIHR: National Institute for Health Research
