# Supplementary material for: Perceptions on the Use of Wearable Sensors and Continuous Monitoring in Surgical Patients: Interview Study Among Surgical Staff
Source: JMIR Form Res. 2022 Feb 11;6(2):e27866. doi: 10.2196/27866 (PMC8881779; doi:10.2196/27866)
Supplement: Multimedia Appendix 1 [file formative_v6i2e27866_app1.docx]

Table S1. The demographics of staff interviewed and years of experience.

| Participant | Consultant Time in healthcare | Consultant Gender | Consultant Education |
| --- | --- | --- | --- |
| 1 | 13 | M | MBBS, FRCS |
| 2 | 25 | M | MBBS, FRCS, PHD |
| 3 | 14 | M | MBBS, FRCS |
| 4 | 27 | M | MBBS, FRCS |
| 5 | 15 | M | MBBS, FRCS |
| 6 | 12 | M | MBBS, FRCS, PHD |
| 7 | 20 | M | MBBS, FRCS |
| 8 | 44 | M | MBBS, FRCS |
| 9 | 18 | M | MBBS, FRCS |
| 10 | 12 | M | MBBS, FRCS, PHD |
| 11 | 15 | M | MBBS, FRCS |
| 12 | 18 | M | MBBS, FRCS |
| Average | 19 |  |  |
| Participant | Senior Nurse Time in healthcare | Senior Nurse Gender | Senior Nurse Education |
| 1 | 20 | F |  |
| 2 | 10 | F |  |
| 3 | 20 | F |  |
| 4 | 20 | F |  |
| 5 | 18 | F |  |
| 6 | 20 | M |  |
| 7 | 6 | M |  |
| 8 | 6 | F |  |
| 9 | 14 | F |  |
| 10 | 15 | F |  |
| Participant | Senior Nurse Time in healthcare | Senior Nurse Gender | Senior Nurse Education |
| 11 | 16 | F |  |
| 12 | 5 | F |  |
| Average | 14 |  |  |
|  |  |  |  |
| Participant | Junior Dr Time in healthcare | Junior Dr Gender | Junior Dr Education |
| 1 | 1 | M | MBBS |
| 2 | 1 | M | MBBS |
| 3 | 5 | F | MBBS, MRCS |
| 4 | 1 | M | MBBS |
| 5 | 1 | F | MBBS |
| 6 | 5 | F | MBBS, MRCS |
| 7 | 1 | M | MBBS |
| 8 | 1 | M | MBBS |
| 9 | 2 | F | MBBS |
| 10 | 1 | F | MBBS |
| 11 | 3 | M | MBBS |
| 12 | 1 | F | MBBS |
| Average | 2 |  |  |
|  |  |  |  |
| Participant | Junior Nurse Time in healthcare | Junior Nurse Gender | Junior Nurse Education |
| 1 |  | M | GCSE |
| 2 | 10 | F | GCSE |
| 3 | 4 | F | GCSE |
| 4 | 20 | F | GCSE |
| 5 | 3 | M | GCSE |
| 6 | 2 | M | GCSE |
| 7 | 8 | F | GCSE |
| 8 | 3 | F | GCSE |
| 9 | 17 | F | GCSE |
| 10 | 19 | F | GCSE |
| 11 | 13 | F | GCSE |
| 12 | 1 | F | GCSE |
| Average | 9 |  |  |
